# The effects of ants on pest control: a meta-analysis

**DOI:** 10.1098/rspb.2022.1316

**Published:** 2022-08-31

**Authors:** Diego V. Anjos, Alejandro Tena, Arleu Barbosa Viana-Junior, Raquel L. Carvalho, Helena Torezan-Silingardi, Kleber Del-Claro, Ivette Perfecto

**Affiliations:** ^1^ Instituto de Biologia, Universidade Federal de Uberlândia, Uberlândia, Minas Gerais 38405-302, Brazil; ^2^ Centro de Protección Vegetal y Biotecnología, Instituto Valenciano de Investigaciones Agrarias (IVIA), Moncada, Spain; ^3^ Programa de Pós-Graduação em Biodiversidade e Evolução, Coordenação de Zoologia, Museu Paraense Emílio Goeldi, Belém, Para 66077-830, Brazil; ^4^ Instituto de Estudos Avançados, Universidade de São Paulo, São Paulo, 05508-020, Brazil; ^5^ School for Environment and Sustainability, University of Michigan, Ann Arbor, MI 48109, USA

**Keywords:** beneficial insects, crop management, herbivory, productivity, sustainability

## Abstract

Environmental impacts of conventional agriculture have generated interest in sustainable agriculture. Biological pest control is a fundamental tool, and ants are key players providing ecological services, as well as some disservices. We have used a meta-analytical approach to investigate the contribution of ants to biological control, considering their effects on pest and natural enemy abundance, plant damage and crop yield. We also evaluated whether the effects of ants are modulated by traits of ants, pests and other natural enemies, as well as by field size, crop system and experiment duration. Overall (considering all meta-analyses), from 52 studies on 17 different crops, we found that ants decrease the abundance of non-honeydew-producing pests, decrease plant damage and increase crop yield (services). In addition, ants decrease the abundance of natural enemies, mainly the generalist ones, and increase honeydew-producing pest abundance (disservices). We show that the pest control and plant protection provided by ants are boosted in shaded crops compared to monocultures. Furthermore, ants increase crop yield in shaded crops, and this effect increases with time. Finally, we bring new insights such as the importance of shaded crops to ant services, providing a good tool for farmers and stakeholders considering sustainable farming practices.

## 1. Introduction

The rapid evolution of pesticide resistance and the risks pesticides pose to human and ecosystem health call for sustainable agricultural practices [1,2]. Biological control of pests is a promising tool in which natural enemies regulate pest densities and reduce damages [3]. Biological control (e.g. providing natural enemies in the ecosystem) not only reduces the use of pesticides and production costs but also helps to maintain local biodiversity [4]. However, the success of biological control depends on many factors, such as the environmental factors and traits of the species involved [5,6].

The citrus growers in China were pioneers in biological control using ants centuries ago [7]. Over time, these organisms have been used to control pests around the world, such as *Spodoptera exempta* (Walk.) in Kenya [8], forest pests in Canada [9], cocoa pests in Ghana [10], crop pests in Nigeria [11] and many other pests in different countries [12,13]. Despite the gradual evolution over time in the use of ants against pests, a major challenge still is to identify positive and negative ant–crop matches, using management to boost positive effects (services) and decrease negative effects (disservices) [14].

Ants are considered as natural enemies of arthropods because they are abundant generalist predators [14–18]. As predators, ants perform services on crops such as reducing pest abundance and plant damage (e.g. lost leaf area, fruit and seed damage), leading to an increase in crop yield [18,19]. However, the role of ants in agriculture is not yet completely clear because they can also cause disservices [20]. For example, ants can spread pathogens, increase the density of honeydew-producing pest species (e.g. mealybugs, soft scales, aphids, psyllids or whiteflies among others), and reduce the abundance of other natural enemies and pollinators [18,20]. For instance, pollinators are able to detect and avoid flowers if ants are present, decreasing pollination services and compromising fruit formation [21,22]. Therefore, it is essential to understand the net effects of ants on biological control.

The main biotic traits driving the role of ants in the biological control of arthropod pests are related to the biology of the species involved (i.e. ants, pests and natural enemies). For instance, aggressive ant species, which are usually abundant and/or large-bodied ant species, are expected to have a greater capacity to reduce pest abundance, mainly non-honeydew-producing ones [14,17]. This reduction can lead to a decrease in plant damage and an increase in crop yield [23–25]. By contrast, these large-bodied or aggressive ants can also affect negatively the abundance and behaviour of natural enemies of honeydew-producing pests [26,27]. The specialization and dispersal type (winged or wingless species) of these natural enemies may be affected by ants due to the lack of elaborate defence mechanisms (most non-parasitoid species) and locomotion capacity of these enemies, respectively [28]. Therefore, it can lead to an increase in honeydew-producing pests [29–31], often causing disservices to agroecosystems [18,20,32].

Besides biotic traits, other factors such as field size, crop system and ant exposure time (i.e. experiment duration) might affect their ecosystem services on biological control in agriculture [33,34]. For instance, the effect of field size on pest density depends on the pest and natural enemy biology but how field size as well crop system might interact with ant services have rarely been evaluated [35]. For instance, more conservative farming with less intensive management (e.g. shaded crops) is expected to conserve or even increase ant diversity and may reflect positively on ants services such as herbivore predation [36,37]. Finally, numerous studies have evaluated the effect of ant exclusion on pest and other natural enemy densities but the experiment duration may have contrasting effects on pest abundance [34] and consequently in plant damage and crop yield.

Despite some meta-analyses testing ant-plant protection interactions [38,39], these studies focus on natural systems rather than agriculture ones. Moreover, with the ongoing increase in the conversion of natural systems into cultivated land [40], we need to understand how biological and environmental traits drive these ant-plant outcomes in agricultural systems. Here, our main goal was to review the role of ants in biological control, considering their services and disservices. To do this, we evaluated the impact of ants on (i) pest abundance, (ii) natural enemy abundance, (iii) plant damage and (iv) crop yield. We used a meta-analytic approach, gathering information from published papers that compared the impacts of ant presence in crops. We also evaluated whether ant effects were modulated by biotic traits, namely: (1) body length of candidate ants (i.e. most abundant species); (2) pest type (honeydew-producing species versus non-honeydew-producing species); (3) pest group (taxonomic level of non-honeydew-producing species); (4) natural enemy specialization (specialist versus generalist) and (5) natural enemy dispersal type (winged versus wingless). In addition, we evaluated whether ant effects were modulated by: (6) field size; (7) crop system (monoculture, intercropped or shaded crops) and (8) experiment duration (e.g. ant exclusion experiment). Our hypotheses of how these traits can affect biological control are presented in table 1. Overall, we hypothesized that ant presence (especially large-bodied ants) would decrease the abundance of non-honeydew-producing pests, mainly those with low locomotion and less defense (e.g. caterpillars, immature insects), leading to decreased plant damage and increased crop yield. We also expected ant presence (and those with large bodies) would decrease the abundance of natural enemies of honeydew-producing pests without affecting the abundance of natural enemies of other pests. In general, we expected ants to decrease the abundance of generalist and wingless natural enemies of pests (compared to specialist and winged natural enemies) due to traits related to defense and locomotion, respectively. Finally, we hypothesized that field size, crop system and experiment duration would modulate the effects of ants on the abundance of pests and their natural enemies, plant damage and crop yield.

**Table 1.** General expected effects of ant presence in biological control. (+) symbols represent positive effects (increase or gain), (–) symbols represent negative effects (decrease or losses), (*) symbols represent neutral effects and NA represents non-evaluated effects.

interacting factors	(i) abundance of pests	(ii) abundance of natural enemies	(iii) plant damage	(iv) crop yield
overall	(−)	(−)	(−)	(+)
ant size	(−) larger ants	(−) larger ants	(−) larger ants	(+) larger ants
	(*) smaller ants	(*) smaller ants	(*) smaller ants	(*) smaller ants
pest type	(+) honeydew-producing	(−) honeydew-producing	(+) honeydew-producing	(−) honeydew-producing
	(−) non-honeydew-producing	(*) non-honeydew-producing	(−) non-honeydew-producing	(+) non-honeydew-producing
pest group	(+) honeydew-producing	(−) honeydew-producing	(+) honeydew-producing	(−) honeydew-producing
	(−) dipterans and lepidopterans	(−) dipterans and lepidopterans	(−) dipterans and lepidopterans	(+) dipterans and lepidopterans
	(*) other groups	(*) other groups	(*) other groups	(*) other groups
natural enemies specialization	NA	(−) generalist	NA	NA
		(*) specialists		
natural enemies dispersal	NA	(−) wingless/(*) winged	NA	NA
field size	(−)	(−)	(−)	(+)
crop system	(*) monocultures	(*) monocultures	(*) monocultures	(*) monocultures
	(*) intercropped	(*) intercropped	(*) intercropped	(*) intercropped
	(−) shaded crops	(−) shaded crops	(−) shaded crops	(+) shaded crops
experiment duration	(−)	(−)	(−)	(+)

## 2. Material and methods

### (a) Data collection and inclusion criteria

We searched for papers in Web of Science and Scopus databases using all available years up to 31st March 2021. We used the preferred reporting items for systematic reviews and meta-analyses protocol for paper search [41]. We used the following key terms in our search: ‘ant' AND ‘biological' AND ‘control'. To complement our dataset, we searched for more studies in recent reviews [14–17,28,38,39,42]. We selected studies according to two criteria. Studies must have: (i) investigated ant effects on the abundance of pests or natural enemies, or effects of ants on plant damage (i.e. damaged leaves or fruit, lost fruit or lost leaf area) or crop yield (i.e. fruit production and fruit biomass); (ii) experimentally evaluated the influence of ants by contrasting ant presence with ant exclusion (e.g. ants have been excluded using physical or chemical barriers) on agrosystems.

Our initial search identified 2682 studies (1207 in Web of Science and 1475 in Scopus) that were potentially appropriate for our review. Of these, 678 were eliminated because they were duplicates and 1953 because they were not about the subject of interest or they were not about studies that compared the ant presence and exclusion protocols (electronic supplementary material, figure S1). Then, after applying our initial inclusion criteria (i, ii), 52 studies from our search remained in our dataset (electronic supplementary material, table S1). Overall, these studies provided 857 cases (gathered through effect size estimates) for our analyses. Despite the existence of pioneering studies (see [9–13]), studies prior to 1987 that were located did not meet the inclusion criteria. Therefore, our review included studies carried out in the last 35 years. Our analyses performed adequately according to the proposed meta-analysis, since we had more than 10 effect sizes for each moderator included in our models [43,44].

### (b) Data extraction and effect sizes

From each selected paper, we recorded the mean abundance, the variance and the sample size of evaluated variables (e.g. abundance of pests and natural enemies, plant damage and crop yield) in which ants were present in the trees/shrubs/herbs of crop (hereafter, control) and in which ants were excluded (hereafter, treatment). Where results were presented graphically, we extracted the mean and variation (e.g. standard error, standard deviance) from the figure using ImageJ. We used Hedges' *g* (J-corrected form) since this dataset had a small sample size [45]. To calculate the Hedges' *g*, we used the ant exclusion treatments as reference groups from which we subtracted the mean values of the control groups (ants present on trees, shrubs or herbs of crops). Negative values of Hedges' *g* thus refer to effect sizes where ant presence reduced the abundance of pests, natural enemies, plant damage, in addition, it is also refers to effect sizes where ants reduce the crop yields.

Most of the studies (90%) were conducted on perennial woody crops, with the exceptions of soya bean, cauliflower, cotton and sweet potatoes, which are non-woody and generally cropped as annual. We extracted information about the most abundant ant species in each study. In addition, we used the Global Ant Database [46] and additional literature to assess ant body length (hereafter, ‘ant size') (electronic supplementary material, table S2). It is worth considering that the size of the most abundant ant is a proxy for one important trait of this organism among many others. Arthropod pests were classified as honeydew producers or non-producers (i.e. pest type). When information about honeydew production was not available within original studies, we searched for such information in the available literature using the pest species name as keyword. Although the red scale *Aonidiella aurantii* Maskell, 1879 (Hemiptera: Diaspididae) does not excrete honeydew, this sessile species has been reported to increase in population size in association with ants that tend honeydew-producing species in the same plant organ [47,48]. Therefore, we classified this species as ‘honeydew-producing'. Moreover, we also classified non-honeydew-producing pests into groups considering their taxonomic level (hereafter, ‘pest group'): Coleoptera, Lepidoptera, Hemiptera, Diptera and phytopathogen (e.g. fungi). Orders with few cases (less than 4) (e.g. Orthoptera, Thysanoptera) were excluded from the analyses. We also excluded effect sizes when the authors evaluated more than one dominant pest species in the same study and did not discriminate which one was considered in the analyses.

We classified the type of dispersal of natural enemies and their level of specialization. The enemy dispersal type (winged versus wingless) was based on the stage of development of individuals considered in each study. For example, coccinellid larvae were considered wingless and adults winged. We considered specialized natural enemies to include parasitoid wasps (Hymenoptera) and predators with extremely specialized pest predation behaviour (e.g. *Scymnus posticalis* Sicard, 1913 (Coleoptera: Coccinellidae); or *Episyrphus balteatus* De Geer, 1776 (Diptera: Syrphidae)) (see [49]). By contrast, natural enemies without specialized predation behaviour were considered generalists. As *Mataeomera dubia* Butler, 1886 (Lepidoptera: Noctuidae) preys upon black scale insects [50], we have considered it a natural enemy.

The field size (hectares of the field where an experiment was carried out), crop system and experiment duration (days) were extracted from each paper. For woody crops, we classified the crop system according to the levels of plant diversity. For instance, when there was only one plant species grown, we classified the system as a monoculture. When the main crop was combined with another plant species, we considered as intercropped (e.g. banana intercropped with coffee tree). Finally, when there were many plant species covering the crops we classified the system as shaded crops *sensu* Perfecto *et al*. [51].

### (c) Statistical analysis

We built four multilevel meta-analytic models (one for each of the following variables: pest and natural enemy abundance, plant damage and crop yield) using the ‘rma.mv' function of the ‘metafor’ package [52] on R software [53]. We used the study identity and the effect size inside each study as random factors to estimate the variation between and within studies, respectively. We considered the overall effect size of ants (without moderators) significantly different from zero if 95% confidential intervals (CI) did not include zero [45]. Finally, we performed separate multilevel mixed-effects models with each ant size, pest type, pest group, enemy specialization and dispersal, field size, crop system and experiment duration as moderators to investigate how these factors separately can modulate the effect size of ants on the main variables of biological control.

To explore the possibility of publication bias we used funnel plots (graphical approach) and Egger's regression test (statistical approach) [54], modified according to Habeck & Schultz [55]. In Egger's regression test, we maintained the same structure of the model evaluating the effect of ants on abundance of pests, for instance, but included the variances of Hedges' *g* as a moderator. If the intercept of the regression test significantly deviated from zero, the overall relationship between the effect size and its respective variance in each study is considered asymmetrical, therefore, biased [56]. Since our dataset consists of a relatively small number of studies, we based our evidence of publication asymmetries on *p* < 0.1 [54]. In addition, we estimated the effect size heterogeneity in the models using *I*^2^. The *I*^2^ statistic describes the percentage of variation across studies due to data heterogeneity rather than chance [57].

In the three main variables (abundance of natural enemies, plant damage and crop yield), we found evidence for a publication bias in our dataset, as shown by both the funnel plots and the intercept of Egger's regression (electronic supplementary material, figure S2). There were higher (for pest abundance and plant damage analysis; *I*^2^ = 87.35% and *I*^2^ = 90.56%, respectively) and moderate (for natural enemy abundance and crop yield analysis; *I*^2^ = 70.14% and *I*^2^ = 68.24, respectively) levels of heterogeneity in our models [58]. Such bias is likely to be associated with outlier comparisons (natural enemies' abundance: five outliers; plant damage: 12; and Crop yield: 11) (through the funnel plot, electronic supplementary material, figure S2) as it seems to disappear when those are removed (see [59,60]). Therefore, we obtained an unbiased dataset according to Egger's regression model [58] but the models' heterogeneity was weakly reduced, especially for those with higher levels of heterogeneity (electronic supplementary material, table S3). The removal of the outliers led to the same qualitative result in most analyses, with differences found only in plant damage analyses considering crop system and pest order as moderators and in the overall effect of ants on crop yield (electronic supplementary material, table S4). Below, we present all results using our unbiased datasets (electronic supplementary material, appendix S1 and table S1).

## 3. Results

### (a) Effects of ants on pest abundance

**Figure 1.**
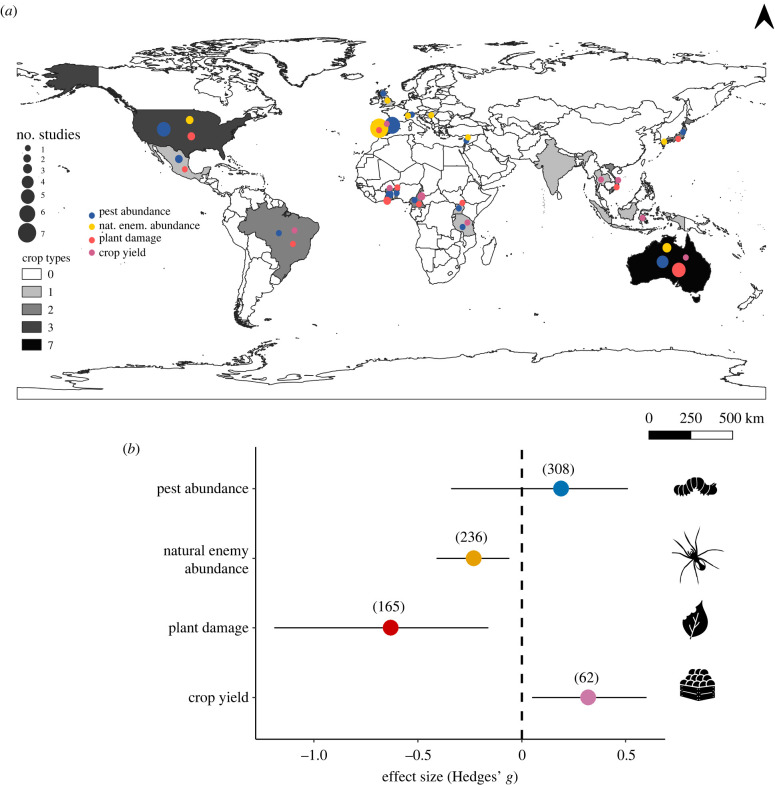
(*a*) Global distribution of the number of studies considering the effects of ants on the abundance of pests and natural enemies, plant damage and crop yield. Dots indicate the country and dot size represents the number of studies. The grey gradient represents the total number of different crops included in all databases. (*b*) Overall effect of ants on the abundance of pests and natural enemies, plant damage and crop yield. Effect sizes and 95% confidence intervals are shown. In parenthesis, the number of effect sizes included in each of the analyses separately. (Online version in colour.)

Our dataset (308 cases) for the effect of ants on pest abundance included 28 studies which comprise 15 countries (figure 1*a*), 26 ant species (from 16 genera) and 30 pest species. The California red scale *A. aurantii* (Hemiptera: Diaspididae) (84 cases), brown citrus aphid *Toxoptera citricidus* Kirkaldy, 1907 (Hemiptera: Aphididae) (38) and the soya bean aphid *Aphis glycines* Matsumura, 1917 (Hemiptera: Aphididae) (16) were the most studied pest species. Most pest species were honeydew-producing (220 cases) compared to non-producing ones (72). Thirteen different crops were assessed, with citrus (169 cases), mango (22), apple and cocoa (21 cases each) crops being the most abundant. *Pheidole pallidula* Nylander, 1849 (Myrmicinae) (62 cases), *Lasius niger* (Linnaeus, 1758) (Formicinae) (49) and *Lasius grandis* Forel, 1909 (Formicinae) (39) were the most abundant ants in the dataset.

**Figure 2.**
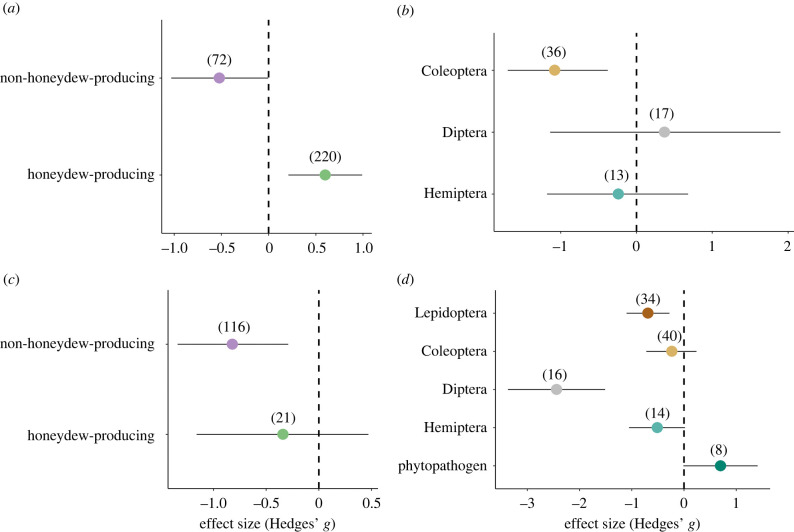
(*a*,*b*) Effect of ants on pest abundance considering (*a*) pest type (honeydew-producing or non-honeydew-producing) (*b*) and pest group. (*c*,*d*) Effect of ants on plant damage considering (*c*) pest type and (*d*) pest group. Effect sizes and 95% confidence intervals are shown. In parenthesis, the number of effect sizes included in each of the analyses, note that the *x*-axis scales are different for (*a*), (*b*), (*c*) and (*d*). (Online version in colour.)

We found a non-significant overall effect of ant presence on pest abundance (Hedges' *g* = 0.19; CI = [−0.17, 0.56]; *p* = 0.31) (figure 1*b*). However, the effect of ants on pest abundance was modulated by pest type (*Q*_M_ = 14.29, d.f. = 1, *p* < 0.01, *n* = 292), showing that ant presence decreased the abundance of non-honeydew-producing species (Hedges' *g* *=* −0.52; CI = [−1.03, −0.005]; *p* < 0.01) and increased the abundance of honeydew-producing ones (Hedges’ *g* *=* 0.60; CI = [0.21, 0.99]; *p* = 0.04) (figure 2*a*). The effect of ants on pest abundance was not significant considering the pest group (*Q*_M_ = 5.51, d.f. = 2, *p* = 0.06, *n* = 66) (figure 2*b*).

### (b) Effects of ants on non-honeydew-producing pests

**Figure 3.**
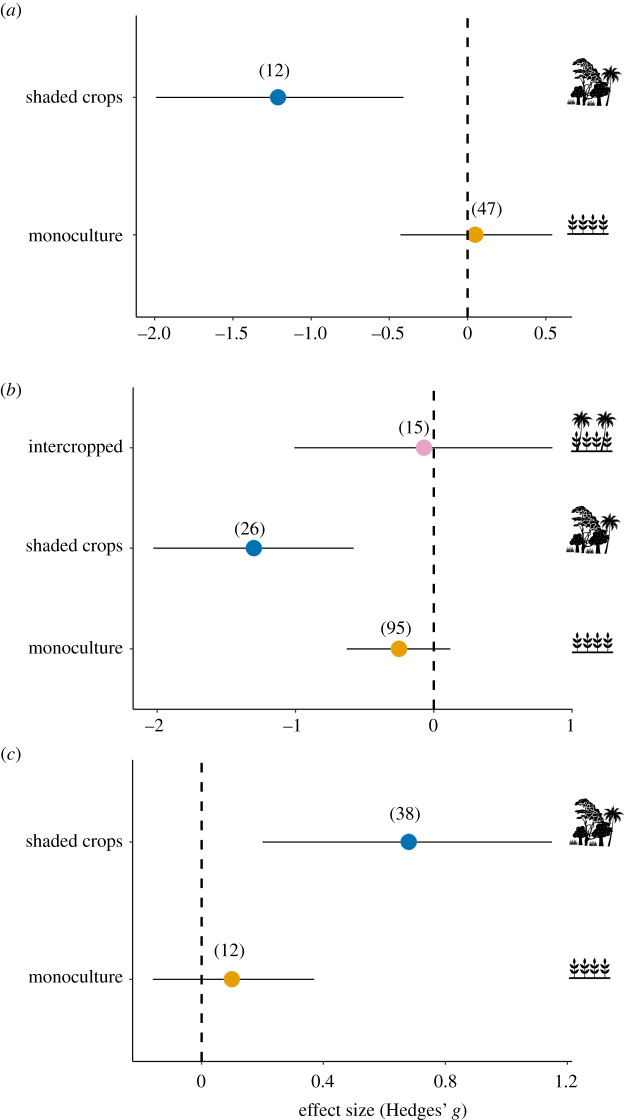
Effect of ants on (*a*) abundance of non-honeydew-producing pests, (*b*) plant damage and (*c*) crop yield analysed by crop system. Effect sizes and 95% confidence intervals are shown. In parentheses are the number of cases included in each of the analyses; note that the *x*-axis scales are different for (*a*), (*b*) and (*c*). (Online version in colour.)

For non-honeydew-producing pests, we found that the effect of ants on pest abundance was not modulated by ant size and experiment duration (electronic supplementary material, table S4). As we found only three field size classes, it was not possible to test this moderator effect. Finally, the crop system modulated the effect of ant presence on the abundance of non-honeydew-producing pests. Ant presence reduced the abundance of these pests on shaded crops but not in monocultures (figure 3*a*; electronic supplementary material, table S4), being that there were no intercropped crops for this analysis.

### (c) Effects of ants on honeydew-producing pests

We found no effects of ants on the abundance of honeydew-producing pests considering the ant size, field size and experiment duration (electronic supplementary material, table S4). For honeydew-producing pests, it was not possible to test the effect of the crop system because all cases were monocultures.

### (d) Effects of ants on natural enemies of pests

The natural enemies dataset (236 cases) included 16 studies, comprising eight countries, nine ant species (from seven genera) and 87 species of natural enemies of pests (figure 1*a*). Six different crops were assessed, with citrus (152 cases), apple (53) and cherry crops (13) being the most abundant. Hymenoptera (49 cases), Coleoptera (45) and Arachnida (29) were the most studied orders of natural enemies. *Lasius niger* (66 cases), *L. grandis* (57) and *Iridomyrmex rufoniger* (Lowne, 1865) (Dolichoderinae) (25) were the most studied ant species. We found a significant negative overall effect of ant presence on the abundance of natural enemies (Hedges' *g* = −0.23; CI = [−0.41, −0.06]; *p* < 0.01) (figure 1*b*), regardless of the pest type of the natural enemy (i.e. non-honeydew-producing or honeydew-producing ones) (electronic supplementary material, table S4).

**Figure 4.**
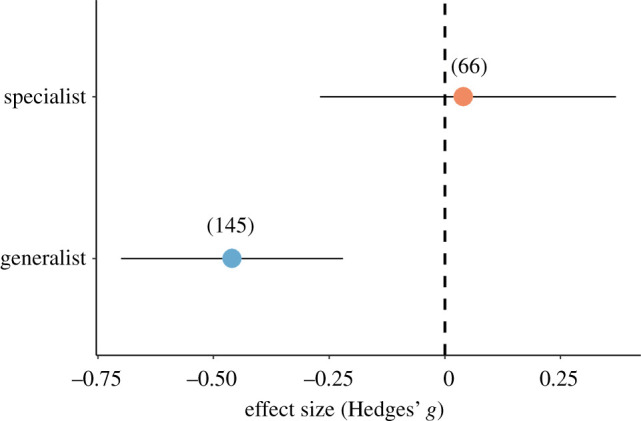
Effect of ants considering the impact of pest specialization on natural enemy abundance. Effect sizes and 95% confidence intervals are shown. In parenthesis, the number of effect sizes included in each of the analyses.(Online version in colour.)

We found no effects of ants on the abundance of natural enemies considering the ant size, pest type, field size and experiment duration (electronic supplementary material, table S4). We also found no effect of ants on enemy abundance considering the enemy dispersal (winged: Hedges' *g* *=* *−*0.20; CI = [−0.40, −0.001]; wingless: Hedges' *g* *=* *−*0.21; CI = [−0.44, 0.02]; *p* = 0.93). However, the enemy specialization modulated the effects of ants on enemy abundance, with ants having a significant negative effect on generalist but not on specialist natural enemies (figure 4; electronic supplementary material, table S4).

### (e) Effects of ants on plant damage

The plant damage dataset (165 cases) included 15 studies, which comprise 13 countries, 15 ant species (from ten different genera) and 10 different crops (figure 1*a*). Cocoa (35 cases), coffee (31) and citrus (30) were the most abundant crops in this dataset. *Oecophylla longinoda* Latreille, 1802 (Formicinae) (40 cases) and *O. smaragdina* Fabricius, 1775 (26)*, L. grandis* and *P*. *pallidula* (seven cases each) were the most abundant ants considering ant effects on plant damage. We found a significant negative overall effect of ant presence on plant damage caused by pests (Hedges' *g* = −0.63; CI = [−1.09, −0.16]; *p* < 0.01) (figure 1*b*).

We found no effects of ants on plant damage considering the ant size, field size and the experiment duration (electronic supplementary material, table S4). We also found no significant effect of ants on plant damage considering the pest type (figure 2*c*), but considering the pest group there was a significant effect (figure 2*d*). Ants decreased plant damage when the pests belonged to the orders Diptera and Lepidoptera. By contrast, ant presence had no effects on plant damage caused by Coleoptera, Hemiptera and phytopathogens (figure 2*d*). Finally, we found that the crop system modulated the effect of ant presence on plant damage. Ant presence reduced the plant damage in shaded crops (Hedges' *g* *=* *−*1.30; CI = [−2.03, −0.58]) but not in intercropped (Hedges' *g* *=* *−*0.07; CI = [−1.01, 0.86]) and monocultures (Hedges' *g* *=* *−*0.25; CI = [−0.63, 0.12]) (figure 3*b*; electronic supplementary material, table S4). Considering the analyses with outliers, the shaded crops cease to be significant because of the cases coming from Dwomoh *et al*. [61], which account for 11 of the 12 outliers found. This analysis may suffer from a methodological bias, for instance, which we were unable to detect.

### (f) Effects of ants on crop yield

Our dataset for crop yield (62 cases) included 10 studies, which comprise nine countries, 11 ant species (from nine different genera) in seven different crops (figure 1*a*). Coffee (24 cases), citrus (17) and cocoa (15) were the most abundant crops. *Oecophylla longinoda* (16)*, L. grandis* (5) and *O. smaragdina* (4) were the most represented ants in these studies. We found a significant positive overall effect of ant presence on crop yield (Hedges' *g* = 0.32; CI = [0.05, 0.60]; *p* = 0.02) (figure 1*b*; electronic supplementary material, table S4). It is important to consider that this latter result and the others below should be interpreted with caution due to the small sample size.

**Figure 5.**
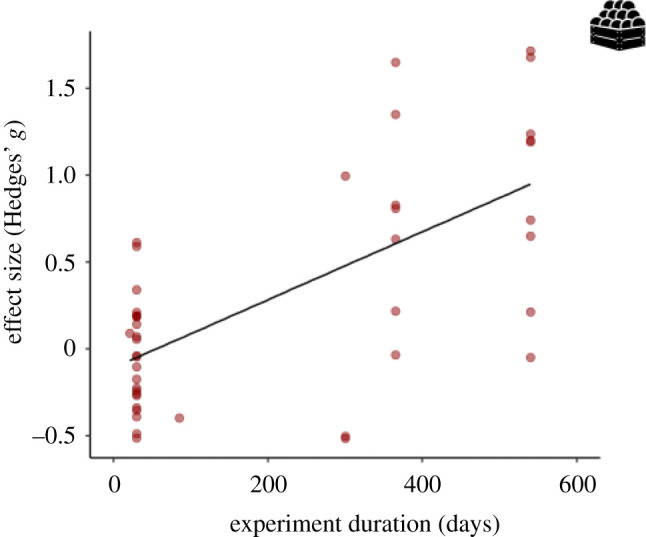
Effect of ants on crop yield (*n* = 45, from six studies) analysed by the duration of experiment. Results were obtained with single meta-regressions. (Online version in colour.)

We found no effects of ants on crop yield considering the ant size (electronic supplementary material, table S4). The experiment duration significantly increased the positive effects of ants on crop yield (figure 5; electronic supplementary material, table S4). The crop system modulated the effect of ants on crop yield. Moreover, ant presence increased the crop yield in shaded crops (Hedges' *g* *=* 0.68, [0.20, 1.15]) but not monocultures (Hedges' *g* *=* 0.10, [−0.16, 0.37]) (figure 3*c*). As there were only three cases of intercropped, this category was excluded from this analysis.

## 4. Discussion

Our results indicate that disservices (mean effect size: with absolute values = 0.40) such as the increased abundance of honeydew-producing pests and decreased abundance of natural enemies are outweighed by biological control ecosystem services provided by ants. Overall, the mere presence of ants, regardless of their body size, provided pivotal services for crops (mean effect size: with absolute values = 0.55) such as the decreased abundance of non-honeydew-producing pests, decreased plant damage and increased crop yield corroborating, at least partially, most of our hypotheses.

Ants decrease the abundance of non-honeydew-producing pests while they increase the abundance of honeydew-producing ones. This occurs because non-honeydew-producing pests are a source of protein for ants and do not offer additional food resources [30,62]. Moreover, all non-honeydew-producing pests considered in this study, except *Panonychus citri* (McGregor 1916) (Acari: Tetranychidae), spend part of their life cycle in the soil, where they are often exposed to predation by ants [28,63].

Crop systems that allow greater plant diversity in the environment (e.g. shaded crops) favour the role of ants in the control of non-honeydew-producing pests. In these systems, there may be a dilution of pest resources, allowing better biological control by ants. Compared to monoculture (Hedges' *g* *=* 0.05), ants decreased the abundance of these pests by 104% in shaded crops (Hedges' *g* *=* −1.21) (figure 3*a*). The presence of other plant species and less intensive tilling in shaded crops reduce the use of pesticides and favour biodiversity preservation [64], including that of predatory arthropods such as ants [51,65]. These traits and management practices in shaded crops provide more ant niches (e.g. nesting sites and food resources) which lead to an increase in ant diversity, i.e. more species inhibiting and preying on pests in these environments [36,66,67]. For instance, regarding arboreal ants, a diverse array of twigs (from different plant species) attracts higher abundance and richness of ants than a monospecific collection of twigs [68]. Diverse ant communities can provide protection against a wider range of pests [37]. Finally, it is worth noting that our data mostly considered the ant community as whole and did not allow comparisons of ant community effects versus single ant species.

Overall, our analyses show that ants tend to increase honeydew-producing pests. Ants often establish mutualistic associations with these pests, receiving honeydew and offering protection from predators [18,30,62]. These associations are often considered a disservice by ants on crops mainly through damage caused by honeydew-producing pests and sometimes by inhibition of natural enemies [37,69]. However, the outcomes of these associations for plants are still uncertain (see [27]). An environmentally friendly management practice to interrupt these associations is to offer an alternative source of sugars (on the ground, near the trunk or on the tree branches). This distraction of ants leads to an increase in natural enemy abundance and a reduction in the size of hemipteran colonies [63,70].

One of the major disservices of ants in crops is to deter or prey on natural enemies of pests (intra-guild predation). Overall, ants consistently decreased the abundance of natural enemies, regardless of the biological traits (i.e. ants, pests and natural enemies), field size and experiment duration. For instance, pest type (honeydew-producing or not), ant size or enemy dispersal (i.e. winged or wingless) did not affect the role of ants, contrary to our initial hypotheses. However, ants decreased the abundance of generalist natural enemies (Hedges' *g* *=* −0.37) by up to 95% compared to specialists (Hedges' *g* *=* −0.02). Specialist natural enemies (specialist predators and parasitoids) have evolved some adaptations to deal with ants, such as chemical camouflage [49]. For instance, Mouratidis *et al*. [71] have recently reported that parasitoids of mealybugs use cuticular hydrocarbons left by ants to avoid mutualistic ants. The effects of ants on natural enemies may be, therefore, less harmful since the simultaneous use of generalist and specialist natural enemies often enhances the effect of these on biological control [72]. This simultaneous effect might partially explain why ant presence reduced plant damage even as the number of natural enemies decreased.

The ants reduced the plant damage regardless of whether it was caused by non-honeydew-producing pests or honeydew-producing pests (see [38]). When we consider pest groups (only those non-honeydew-producing species), ant presence did not affect any abundance of the groups assessed. However, ants reduced plant damage caused by Diptera and Lepidoptera. Fruit flies (the main group of dipterans studied in this review) avoid laying eggs in fruit with ant pheromones [73,74] and ants prey on fruit flies that pupate in the soil [63]. In addition, dipteran larvae and caterpillars may also be especially vulnerable to ants due to their few defense mechanisms [20,23,75]. Aluja *et al*. [76] found that larvae of *Anastrepha* spp. (Diptera: Tephritidae) on the ground are mostly attacked by ants within 5 min after leaving the fruit. Ants had no effect on plant damage caused by Coleoptera, Hemiptera and phytopathogens. However, this latter result should be interpreted with caution since we had only eight cases of phytopathogens. Finally, the crop system also modulated the effect of ants on plant damage. As ant diversity may increase in plant-richness environments [68] such as shaded crops [67], whereby ants tend to increase the predation rate [36,66]. These effects can directly reflect in the reduction of plant damage and consequently in the increase in crop yield (figure 3*b,c*). Therefore, shaded crops, as well as other crop systems like organic agriculture and agroforestry [64], can enhance the beneficial services provided by ants (e.g. pest control) that increase the economic benefits to farmers.

The crop yield is the ultimate ecosystem service and one of the most important to agricultural systems, mainly from the point of view of farmers. Our meta-analysis shows that ants increase crop yield. In Northern Australia, for instance, economic estimates have shown that the use of top dominant ant, *O. smaragdina*, can increase cashew production by 49%, generating a net income of 70% (including costs and gains from the use of ants instead of chemical insecticides) [77]. The longer the duration of the study the greater the effect sizes on crop yield. Therefore, once ant colonies are established, the benefits to crops tend to increase over time (at least ± 2 years, as shown in our dataset). This may be a key benefit of using ants in biological control because pesticides cannot have effect on some pest species or they develop resistance over time, requiring new pesticides and increasing costs [78,79]. However, we cannot rule out that assessing the effect of ants in the short term may be controversial.

## 5. Conclusion

Our study is the first evidence synthesis investigating ants' primary services and disservices in the pest control of multiple crops. It is worth noting that different ant species act as biological controls in different ways [28]. The services provided by ants outweigh the disservices. Overall, our results corroborate general patterns and hypotheses and bring new insights to the role of ants in biological control. Our meta-analyses show that the effect of ants as biological controls is more pronounced in shaded crops, aligning two major sustainable management practices. The presence of ants in shaded crops can improve pest management and increase crop yield, as well as increase biodiversity within agroecosystems (see [80]). Therefore, from our results, we encourage practices of shaded crops as a way to naturally promote ants in crop systems. In most cases, ants are low-cost solution. However, in others, it is necessary to move colonies into the crop areas and provide food and/or nests for their survival, as occurs with *Oecophylla* ants in many locations worldwide [42]. In general, with proper management, ants can be useful pest controls and increase crop yield over time. Some ant species have similar or higher efficacy than pesticides, at lower costs [14]. Moreover, ants can be used with integrated pest management when ants alone are not enough to control the pest [24]. Finally, further studies investigating other factors that can affect the role of ants on pest control in a changing world, such as landscape composition, climate change and ant invasive status should be encouraged.

## Data Availability

The data supporting the results are archived on Zenodo database [81]. The data are provided in electronic supplementary material [82].
